# Community‐Acquired Pneumonia Manifested by Acute Abdominal Pain: A Case Report

**DOI:** 10.1155/crdi/3797000

**Published:** 2026-06-16

**Authors:** Siwar Belhaj Salem, Ariba Furqan, Ramla Mohamed Farah Roble, Sarah Suwal, Linh Huynh, Syed Muhammad Hassan, Sara S.

**Affiliations:** ^1^ Department of Family Medicine, Faculty of Medicine of Monastir, Monastir, Tunisia, um.rnu.tn; ^2^ Department of Internal Medicine, Dow University of Health Sciences, Karachi, Pakistan, duhs.edu.pk; ^3^ Department of Internal Medicine, School of Medicine Ahfad University for Women, Omdurman, Sudan; ^4^ Department of Internal Medicine, Nepal Medical College and Teaching Hospital, Kathmandu, Nepal; ^5^ Department of Internal Medicine, University of California, Irvine, California, USA, berkeley.edu; ^6^ Department of Internal Medicine, Karachi Medical and Dental College, Karachi, Pakistan; ^7^ Department of Internal Medicine, Government Medical College Ramanathapuram, Ramanathapuram, India

**Keywords:** abdominal pain, case report, community-acquired pneumonia, fluoroquinolones, *Streptococcus pneumoniae*

## Abstract

Pneumonia is a common acute respiratory infection that affects the alveoli and distal airways and typically presents with symptoms such as chest pain, cough, dyspnea, and fever. However, it can also manifest with nonspecific symptoms like abdominal pain. This case report presents a previously healthy 24‐year‐old male who initially presented with severe right upper quadrant abdominal pain and fever. Despite initial treatment, the patient’s condition worsened, with the emergence of dyspnea and productive cough. Subsequent examination and imaging revealed community‐acquired pneumonia (CAP) caused by *Streptococcus pneumoniae*. The diagnosis was confirmed through clinical examination, chest X‐ray showing a right lower lobe infiltrate, and sputum analysis. The patient was successfully treated with intravenous fluoroquinolone and oral antibiotics upon discharge. This case underscores the importance of considering CAP in the differential diagnosis of acute abdominal pain to avoid misdiagnosis and ensure timely treatment. It highlights the need for awareness of atypical presentations of CAP.

## 1. Introduction

Pneumonia is an inflammatory lung condition which primarily affects alveoli, leading to symptoms such as chest pain, dry or productive cough, dyspnea, fever or hypothermia, and sweating, but it may present with nonspecific symptoms especially at extremes of ages, where presenting features may vary such as fatigue, myalgia, abdominal pain, anorexia, and headache. This condition can be of variable severity, making its diagnosis among the mainstays of emergency medicine. Pneumonia can present with extrapulmonary symptoms, and abdominal pain is one of them, occurring in about 8% of patients. In children, pneumonia is the most frequent extra‐abdominal cause of acute abdominal pain. However, this particular association has been subjected to relatively limited examination within the adult population [1, 2]. The diagnosis of pneumonia is based upon clinical symptoms, examination findings, and further verified by imaging. Pneumonia constitutes a prevalent etiological factor for sepsis; therefore, it is imperative to consider the implementation of early and aggressive treatment targeting the most likely organisms in order to mitigate the risk of clinical deterioration [1]. Further attention to atypical community‐acquired pneumonia (CAP) presentation is therefore needed. In this case report, we will report a patient who presented to the emergency department with abdominal pain that revealed pneumonia upon imaging.

## 2. Case Presentation

A 24‐year‐old male presented to the Teaching Hospital of Monastir, Tunisia, with severe abdominal pain in the right upper quadrant (RUQ) and fever. He has no prior medical history, and his vaccinations were up‐to‐date. On examination, the patient was conscious and well oriented. His vital signs were as follows: temperature 39.5°C, blood pressure 120/70 mmHg, pulse rate 80 beats per minute, and respiratory rate 16 breaths per minute. Oxygen saturation was 99%. On abdominal examination, tenderness was present in the RUQ with a VAS of 6 out of 10. There was no rebound tenderness or guarding. Examination of other systems was unremarkable. The onset of the current symptoms was noted one day prior to the patient’s admission to the emergency department. Laboratory tests were ordered based on his findings. A complete blood count showed a hemoglobin level of 13 g/dL, high white blood cell counts of 21,000/μL, and a normal platelet count of 1,65,000/μL. The C‐reactive protein (CRP) level was elevated to 240 mg/L (As shown in Table 1). The renal function test was within normal limits. A noncontrast CT scan of the abdomen and pelvis was performed which did not show any abnormalities. The patient was discharged from the hospital with a presumptive diagnosis of gastroenteritis. The patient returned to the emergency department 48 h later with persistent fever and additional complaints of difficulty in breathing, productive cough, and fatigue. On examination, the patient appeared ill with a temperature of 39°C and respiratory rate of 22 breaths per minute. Oxygen saturation had decreased to 94%. On chest examination, crackles were heard on auscultation over the right lower lung field. No retractions were seen. At the time of admission, severity assessment was performed using the CURB‐65 score. The patient had no confusion, normal blood pressure, respiratory rate of 22 breaths per minute (< 30), and age < 65 years, with normal renal function. This resulted in a CURB‐65 score of 0, indicating low predicted mortality and typically supporting outpatient management.

**TABLE 1:** Laboratory results of the patient.

Test	Result	Reference range	Unit
Hb	13	Males: 13–16; females: 12–14	g/dL
WBC	21,000	4500–11,000	cells/μL
Platelet	165	150–450	10^3^/μL
CRP	240	< 8	mg/L
Urea	5.2	3.3–7.0	mmol/L
Creatinine	65	53–120	μmol/L
AST	6	5–45	UI/L
ALT	8	5–45	UI/L
pH	7.38	7.35–7.45	—
HCO_3_^-^	28	22–28	mmol/L
PCO_2_	48	35–45	mm Hg
PO_2_	72	> 80	mm Hg

*Note:* Hb, hemoglobin; AST, aspartate aminotransferase; ALT, alanine transaminase; HCO_3_, bicarbonates; PCO_2_, partial pressure of carbon dioxide; PO_2_, partial pressure of oxygen.

Abbreviations: CRP, C‐reactive protein; WBC, white blood count.

The patient was then admitted to the hospital. Based on his symptoms, an arterial blood gas (ABG) analysis, chest X‐ray (CXR), sputum test, and blood culture were ordered. ABG analysis revealed hypoxemia with a PaO_2_ of 65 mmHg, PaCO_2_ of 34 mmHg, and pH of 7.46, consistent with mild respiratory alkalosis and impaired oxygenation. Despite a low CURB‐65 score, the presence of hypoxemia suggested more significant pulmonary involvement. This discrepancy between clinical severity score and gas exchange abnormality supported the decision for hospital admission and close monitoring.

**FIGURE 1:**
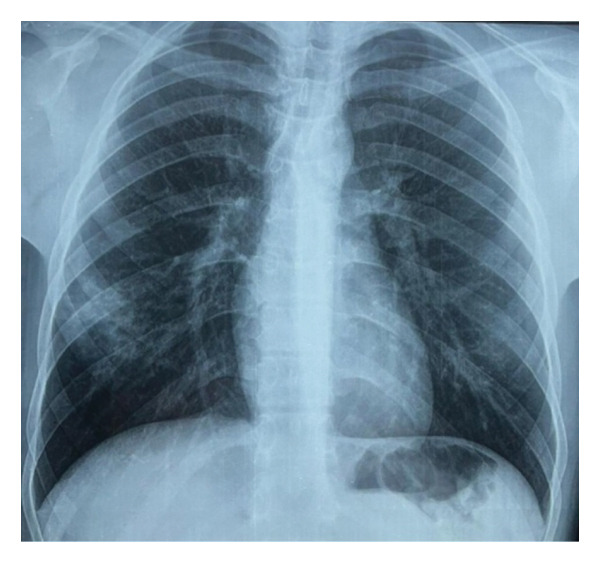
Chest X‐ray of our patient showing an infiltrate of the lower lobe of the right lung.

CXR showed an infiltrate in the right lower lobe (as shown in Figure 1), and sputum showed *Streptococcus pneumoniae*. Blood culture was negative. After a definitive diagnosis of CAP was made, the patient was treated with IV fluoroquinolone (FQ) along with IV fluids and oxygen. Given the presence of hypoxemia requiring hospitalization, empiric therapy with an intravenous respiratory FQ was selected to ensure adequate coverage of both typical and atypical pathogens. The patient showed improvement after treatment and was discharged on the third day with oral antibiotics. Our patient experienced an improvement in both psychological and physical well‐being and has expressed a willingness to return to his daily activities and job.

The authors adhered to the CAse REport (CARE) guidelines (Supporting 1).

## 3. Discussion

This case involves a previously healthy 24‐year‐old male who initially presented with fever and RUQ abdominal pain, which was suggestive of gastroenteritis. However, after comprehensive examination and imaging, CAP was diagnosed, with sputum analysis revealing *Streptococcus pneumoniae*. This case highlights the possibility of CAP presenting with atypical symptoms beyond the lungs, emphasizing the need for vigilance to avoid misdiagnosis.

Pneumonia is a prevalent inflammatory lung disease that may present with various symptoms, including extrapulmonary manifestations such as abdominal pain [2]. Atypical presentations of CAP occur predominantly with abdominal symptoms, especially among children, the elderly, and the immunocompromised [1, 3]. While CAP in adults typically manifests with respiratory symptoms, atypical presentations are less prevalent [4]. Abdominal pain in CAP—particularly in lower lobe pneumonia—should not be overlooked as a potential referred symptom rather than a primary abdominal pathology. This is especially relevant when consolidation is adjacent to the diaphragm. The most plausible mechanism is referred pain due to diaphragmatic or pleural irritation. In lower lobe pneumonia, inflammatory involvement of the parietal pleura can stimulate the phrenic nerve (C3–C5), resulting in perceived pain in the RUQ or epigastrium, which may clinically mimic acute abdominal conditions [5, 6].

Various CAP pathogens may cause extrapulmonary manifestations, including gastrointestinal and dermatological symptoms, with rare cases of hepatitis, acute pancreatitis, and Henoch–Schonlein purpura. Gordon et al. reported that 26% of children with *Mycoplasma pneumoniae* (MP) had extrapulmonary symptoms, with 39% lacking respiratory symptoms [7]. Another case of a 35‐year‐old man with fever, cough, and jaundice was diagnosed with MP infection after excluding common hepatic causes. Treatment led to rapid improvement, highlighting CAP’s potential to cause abdominal symptoms in adults [8]. MP is a respiratory pathogen that adheres to and colonizes the airway epithelium, leading to a range of clinical manifestations including upper respiratory tract infection, bronchitis, bronchiolitis, and pneumonia. In certain cases, the infection may present in an atypical manner, where extrapulmonary features such as abdominal pain, testicular swelling or pain, and fever predominate without significant respiratory symptoms. The organism is believed to cause disease through both direct stimulation of local immune responses and dysregulated inflammation involving monocytes, macrophages, lymphocytes, and epithelial cells, which results in the release of multiple cytokines and subsequent tissue injury. Furthermore, immune complex formation is considered an important mechanism underlying extrapulmonary complications. Gastrointestinal involvement may also be observed, often in the form of nonspecific symptoms, including liver function abnormalities [9]. Similarly, SARS‐CoV‐2 virus has been associated with a wide range of symptoms which cause the syndrome known as COVID‐19*.* COVID‐19 may induce diarrhea, nausea, vomiting, and abdominal pain in up to 50% of patients. Patients may also have elevated LFTs and bilirubin. Pancreatitis‐like presentations have been well documented [1]. *Chlamydia psittaci* is another pathogen to cause CAP with extrapulmonary symptoms. It constitutes a rare respiratory pathogen, responsible for approximately 1% of cases of CAP. Various extrapulmonary complications may occur such as acute liver injury, acute renal failure, myocarditis, and meningitis. Common clinical symptoms of psittacosis include severe pyrexia, chills, headache, shortness of breath, and cough. Few cases complicated by acute pancreatitis have also been reported. It is noteworthy that a variety of pathogens, including SARS‐COV‐2, MP, and *Legionella* spp., may cause acute pancreatitis [10].

The pathogen must be precisely identified, and one should take into account the severity of the symptoms in order to administer the appropriate medication. Oral amoxicillin is the primary empirical treatment for low‐severity CAP [11]. However, our patient presented with severe symptoms, including persistent fever, difficulty breathing, productive cough, and fever, which require a different treatment approach. Therefore, the patient was treated with IV FQ. FQ inhibits bacterial proliferation and has anti‐inflammatory activities [12].

Pneumonia can prove to be a challenging diagnosis, particularly when accompanied by extrapulmonary symptoms. Such presentations are frequently observed in immunocompromised individuals, the elderly, and pediatric patients. Nonetheless, healthy adults may also exhibit similar presentations. *Streptococcus pneumoniae* infrequently induces this type of presentation. Prompt diagnosis can enhance the clinical outcome and avert potential complications.

## 4. Conclusion

CAP can present as acute abdomen as seen in this patient. It is easy to misdiagnose CAP as a gastrointestinal pathology when only abdominal symptoms are present. This can lead to delayed treatment and worsening of patient condition. Thus, it is important to consider pneumonia in the differential diagnosis of abdominal pain in patients presenting with acute abdomen. In particular, lower lobe pneumonia should be recognized as a potential cause of abdominal pain due to referred pain mechanisms. This referred pain is most likely secondary to diaphragmatic or pleural irritation, with transmission via the phrenic nerve leading to pain perceived in the RUQ or epigastric region.

Although it may be challenging, especially in the absence of respiratory symptoms, a thorough evaluation of the patient with relevant history, physical examination, tests, and imaging may help guide the physician toward the correct diagnosis and timely treatment of the patient.

## Author Contributions

Siwar Belhaj Salem and Ramla Mohamed Farah Roble: initial draft, conceptualization, data collection, and writing.

Ariba Furqan, Sarah Suwal, Linh Huynh, Syed Muhammad Hassan, and Sara S.: writing and reviewing.

## Funding

The authors have nothing to report.

## Ethics Statement

Ethical approval was not required for this case report in accordance with the policies of the Faculty of Medicine of Monastir.

## Consent

Written informed consent was obtained from the patient for the publication of this case report and accompanying figures.

## Conflicts of Interest

The authors declare no conflicts of interest.

## Supporting Information

Additional supporting information can be found online in the Supporting Information section.

## Supporting information


**Supporting Information** Supporting 1: The authors adhered to the CAse REport (CARE) guidelines throughout the writing of this manuscript. All relevant checklist items, including patient information, clinical findings, diagnostic assessment, therapeutic intervention, and follow‐up outcomes, were systematically addressed and documented.

## Data Availability

Data are available upon request from the authors.
